# Supplementary material for: Tumor fitness, immune exhaustion and clinical outcomes: impact of immune checkpoint inhibitors
Source: Sci Rep. 2020 Mar 19;10:5062. doi: 10.1038/s41598-020-61992-2 (PMC7081289; doi:10.1038/s41598-020-61992-2)
Supplement: Supplementary file 1 — Supplementary information. [file 41598_2020_61992_MOESM1_ESM.pdf]

Tumor fitness, immune exhaustion and clinical outcomes: impact of immune checkpoint inhibitors

Adrian Bubie[1], Edgar Gonzalez-Kozlova[1], Nicholas Akers[2], Augusto Villanueva[3], Bojan Losic[4]*

1: Department of Genetics and Genomic Sciences, Icahn Institute for Data Science and Genomic Technology, Icahn School of Medicine at Mount Sinai, New York, NY 10029
2: Adaptive biotechnologies, 1551 Eastlake Avenue E, Suite 200, Seattle WA 98102
3: Division of Liver Diseases, Department of Medicine, Liver Cancer Program, Tisch Cancer Institute, Icahn School of Medicine at Mount Sinai, New York, USA.
4: Department of Genetics and Genomic Sciences, Tisch Cancer Institute, Cancer Immunology, Diabetes, Obesity and Metabolism Institute, Icahn Institute for Data Science and Genomic Technology, Icahn School of Medicine at Mount Sinai, New York, NY 10029

*Correspondence to: bojan.losic@mssm.edu

Supplemental Materials:

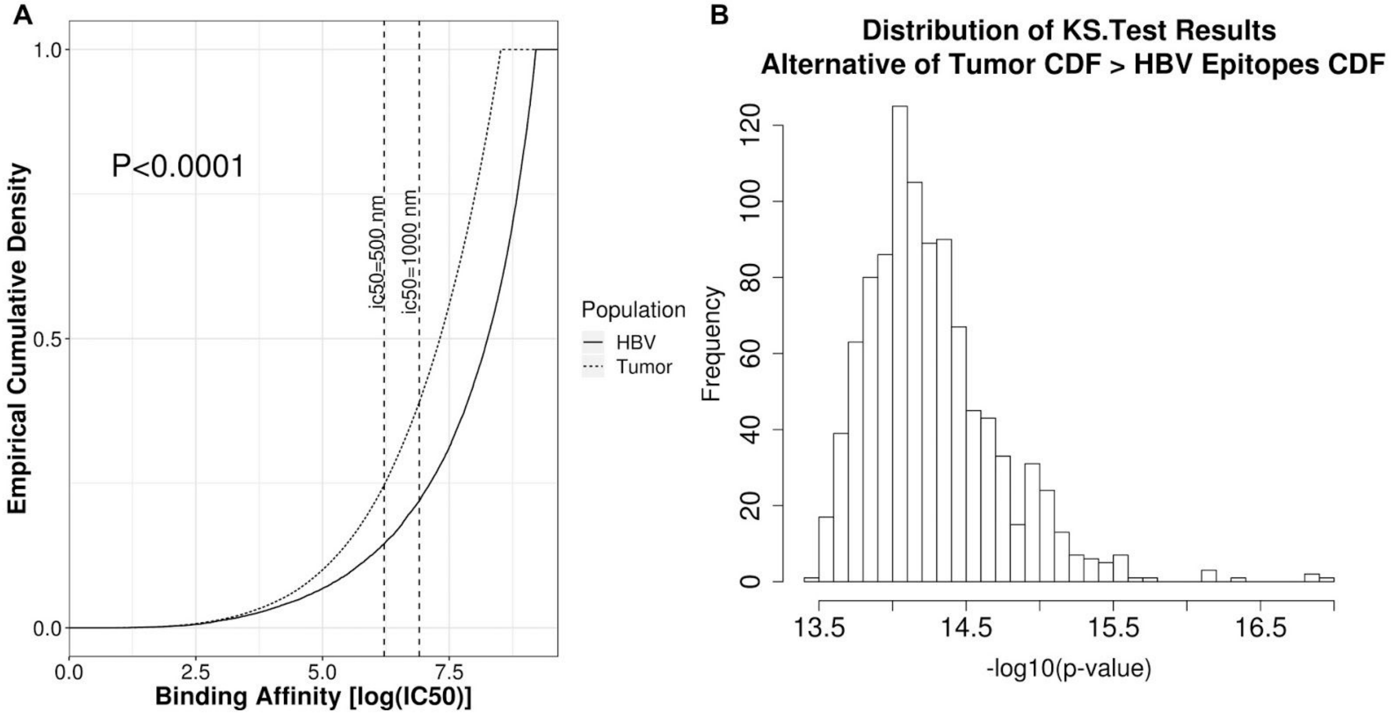

Supp. Figure 1: Kolmogorov-Smirnov test results (**A**) of randomly subsampled distributions of tumor neoantigen binding affinities compared to HBV antigens across 1,000 iterations. **B)** Distribution of test p-values strongly support the alternative hypothesis that tumor neoantigen cumulative distributions were significantly less than HBV distributions, indicating better average binding affinity scores are robust to subsampling.

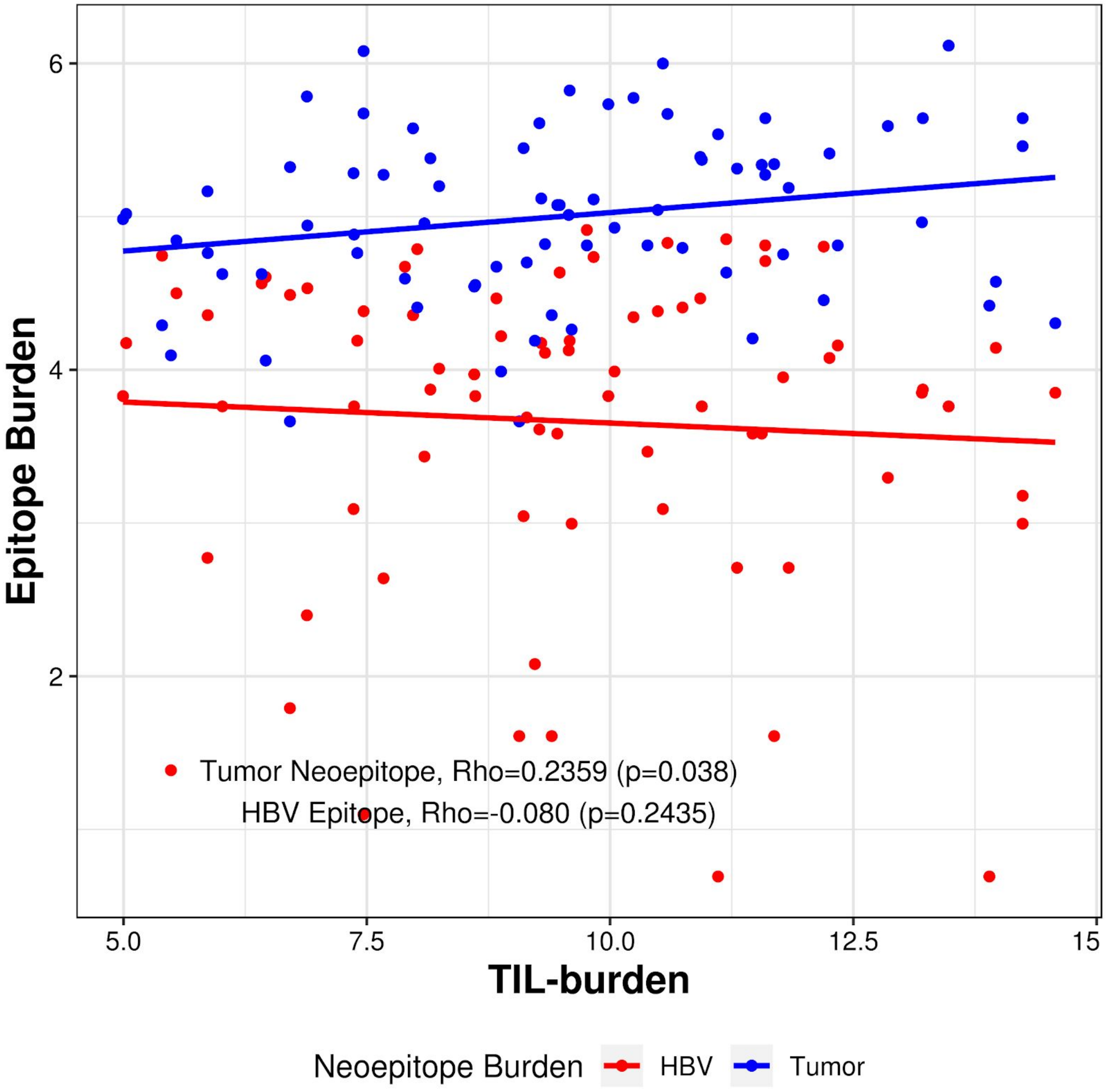

Supp. Figure 2: Tumor neoantigen burden is slightly but significantly positively correlated with patient TIL-burden, while HBV antigen load is not. Spearman correlations are presented for epitope group and TIL-burden associations.

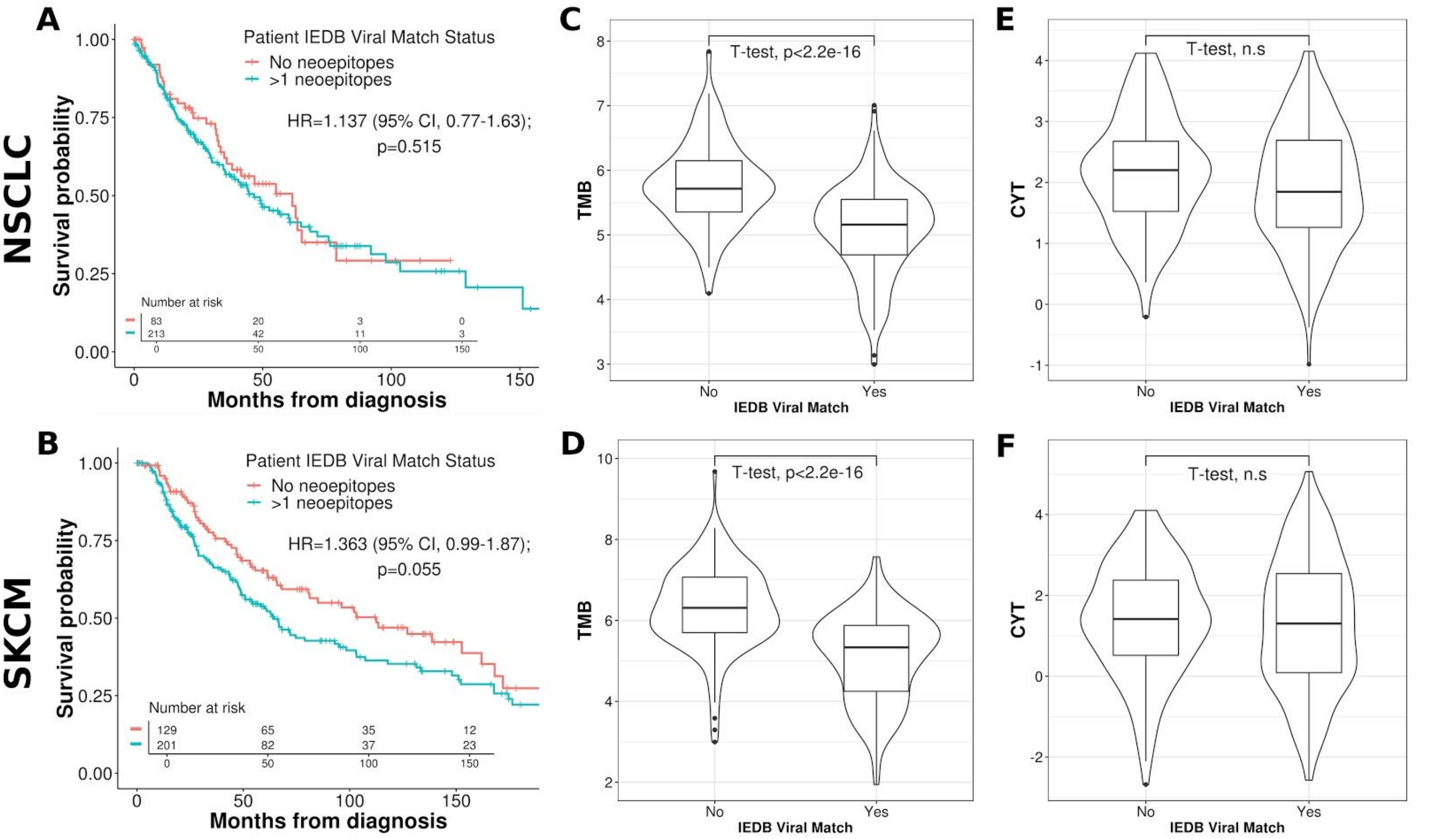

Supp. Figure 3: Kaplan-Meier survival curves between patients with and without neoepitope BLAST matches against the viral IEDB database for the **A)** lung and **B)** melanoma TCGA cohorts. Tumor mutational burdens for patients with viral neoantigen matches fall significantly below those with no matches in both the **C)** lung and, **D)** melanoma patient groups (two-tailed T-test). Differences in cytolytic activity (CYT) expression were found to be negligible for both the NSCLC and SCKM patients between those with and without IEDB matches (**E** and **F**, respectively) (two-tailed T-test).

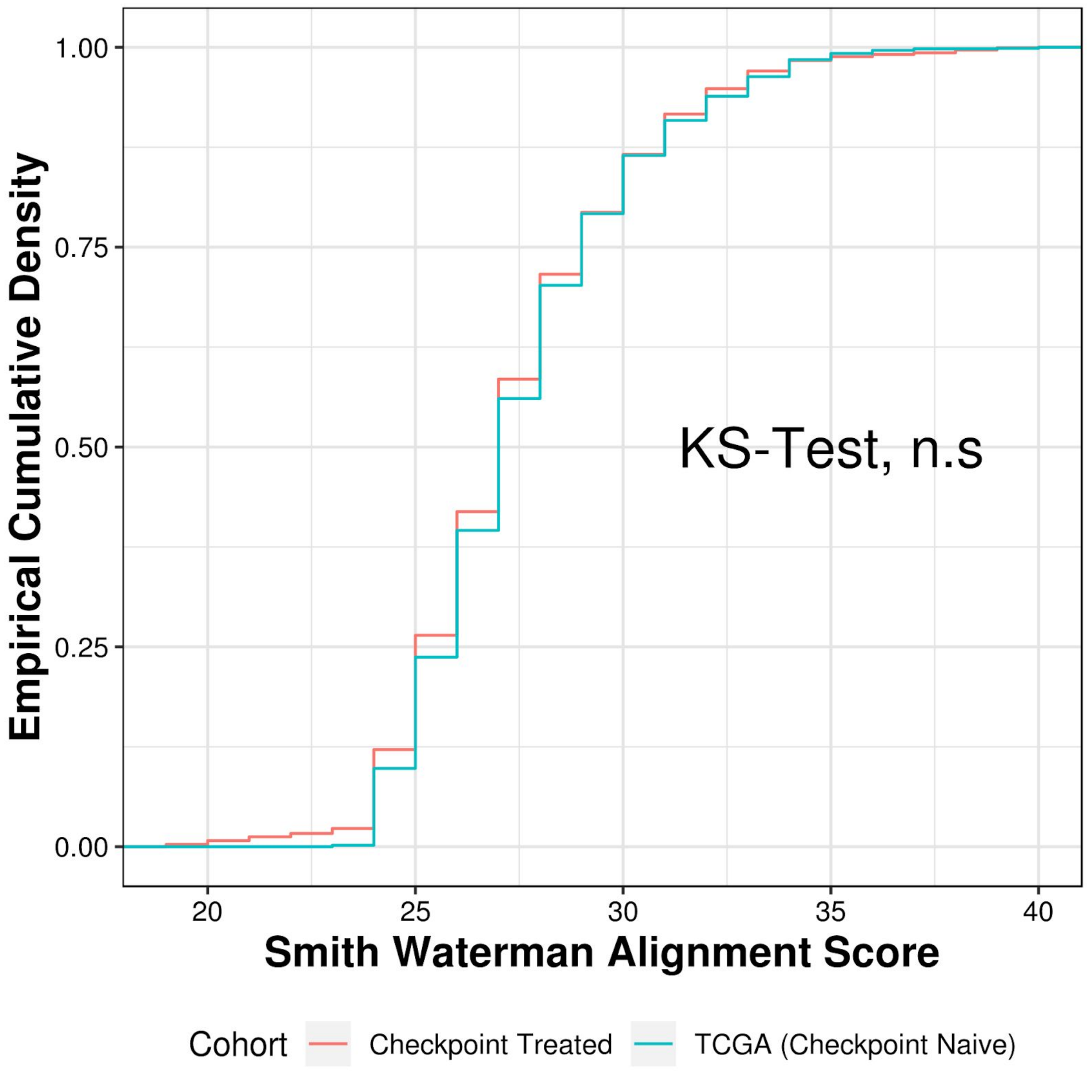

Supp. Figure 4: Comparison of smith-waterman alignment score distributions for tumor neoepitopes to best matching viral epitopes from the TCGA lung and melanoma cohorts and the checkpoint treated patient cohorts used in *Luksza et al*. By Kolmogorov-Smirnov two-sided test, no significant difference between alignment score distributions was observed.

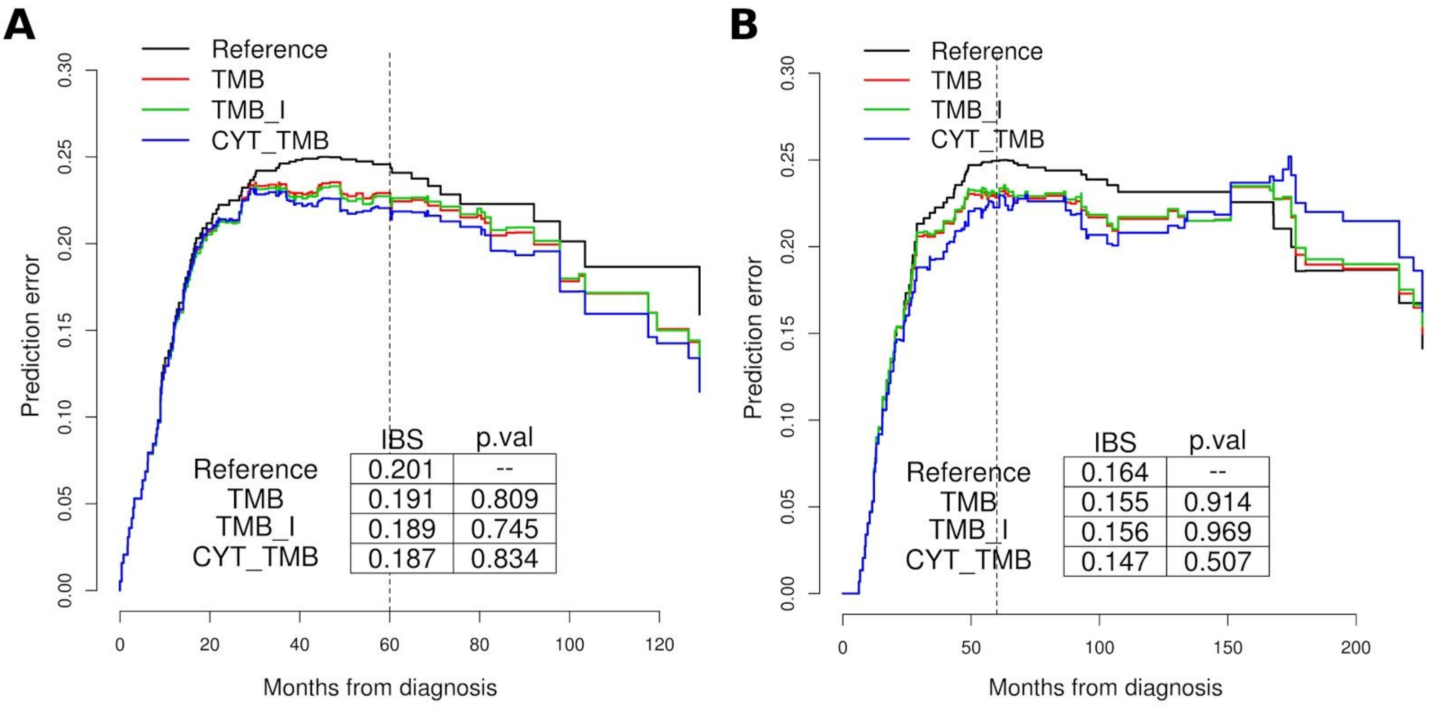

Supp. Figure 5: Prediction error curves for Cox survival models in **A**) lung, **B**) melanoma TCGA cohorts using covariates TMB, TMB and Immunogenicity (I), and TMB and CYT, with respect to months from patient diagnosis. Time dependent integrated Brier scores were evaluated at 5 year survival from diagnosis (60 months). Under this evaluation criteria, no models significantly reduced prediction error over the naive reference model.
